# Stony coral tissue loss disease indirectly alters reef communities

**DOI:** 10.1126/sciadv.adk6808

**Published:** 2024-05-03

**Authors:** Sara D. Swaminathan, Kevin D. Lafferty, Nicole S. Knight, Andrew H. Altieri

**Affiliations:** ^1^ Department of Environmental Engineering Sciences, University of Florida, Gainesville, FL 32611, USA.; ^2^ Western Ecological Research Center, US Geological Survey, Santa Barbara, CA 93455, USA.; ^3^ Marine Science Institute, University of California, Santa Barbara, CA 93106, USA.; ^4^ Department of Biology, McGill University, Montreal, QC H3A 1B1, Canada.; ^5^ Smithsonian Tropical Research Center, Ancon 0843-03092, Republic of Panama.

## Abstract

Many Caribbean coral reefs are near collapse due to various threats. An emerging threat, stony coral tissue loss disease (SCTLD), is spreading across the Western Atlantic and Caribbean. Data from the U.S. Virgin Islands reveal how SCTLD spread has reduced the abundance of susceptible coral and crustose coralline algae and increased cyanobacteria, fire coral, and macroalgae. A Caribbean-wide structural equation model demonstrates versatility in reef fish and associations with rugosity independent of live coral. Model projections suggest that some reef fishes will decline due to SCTLD, with the largest changes on reefs that lose the most susceptible corals and rugosity. Mapping these projected declines in space indicates how the indirect effects of SCTLD range from undetectable to devastating.

## Teaser

Ecological networks within coral reef ecosystems are predicted to affect reef fish responses to stony coral tissue loss disease.

## INTRODUCTION

In less than 10 years, a deadly infectious agent called stony coral tissue loss disease (SCTLD) has emerged as a leading threat to Caribbean coral reefs (*1*–*4*). Although its etiology is not fully understood, the discovery of bacterial disease indicators (*5*, *6*) and the success of probiotic treatments (*7*–*9*) suggest that bacteria are involved. Other evidence suggests that SCTLD is caused by a virus that attacks some corals’ endosymbiotic algae, but not others (*10*, *11*). SCTLD has reduced coral cover by 30 to 60% in affected regions (*2*, *3*), pushing a few coral species toward local extinction (*3*, *12*, *13*). Because diseases are most likely to have indirect community effects when they affect connected, unique, or widespread host species (*14*–*18*), we hypothesized that SCTLD-caused coral loss could trigger broad changes in diverse coral reef ecosystems (*19*).

Although SCTLD represents a grave threat to Caribbean reefs, these ecosystems are not naïve to stress. Early reductions in herbivores due to fishing and disease (*20*, *21*) favored algae over corals (*22*, *23*). Since then, warmer temperatures (*24*) have made bleaching events commonplace (*25*, *26*). Several diseases (*27*–*29*) have further decreased coral cover. The rise of coral-specific diseases in recent decades has led to concerns for the tropical reef organisms that coral reefs support (*30*). For fish, the coral reef’s complex matrix of hard substrate makes ideal shelter from predators or physical disturbances (*31*–*35*) and competitors (*36*). As a consequence, coral loss reduces functional redundancy (*37*), recruitment (*38*), and biomass (*39*) of reef fish communities so that reef fish species richness could decline by 50% if live coral completely disappeared (*40*). Dead coral erodes as a result of ocean acidification, wave action, and bioerosion processes, like grazing and boring (*41*). Because many fish benefit from habitat complexity (*42*–*44*), the effects of SCTLD on fish communities could increase over time as dead coral erodes or decrease as coral populations recover (*41*). Coral loss also affects humans, as coral reefs buffer shorelines from hurricanes (*45*), attract tourism (*46*), and feed coastal communities (*47*). Given these broad concerns for coral reef community composition and physical structure, we investigated the extent to which benthic assemblages (i.e., corals and other dominant space holders) respond to SCTLD and how these changes can affect associated reef fish communities.

We used multilevel Bayesian regression and structural equation modeling to analyze two publicly available survey datasets of benthic and fish communities in SCTLD-affected regions. The first dataset, collected by the Territorial Coral Reef Monitoring Program (TCRMP) in the U.S. Virgin Islands (USVI; *n* = 34 sites), documented the presence and progression of SCTLD over time (January 2019–April 2021), making it suitable for an analysis of the short-term direct impact of SCTLD on coral communities and indirect impacts on benthic communities (*48*). The second dataset, collected by NOAA’s National Coral Reef Monitoring Program (NCRMP), spanned the Dry Tortugas, the USVI, and Puerto Rico (*n* = 1610 sites) (*49*). Because of its size and breadth, the NCRMP dataset was well suited for a Bayesian structural equation model (SEM) assessing relationships between fish and benthic organisms that are most affected by SCTLD. Together, the TCRMP and NCRMP datasets allowed us to (i) capture the impact of SCTLD on benthic and coral communities in detail, (ii) elucidate relationships between coral and fish communities across the broader Caribbean region, and (iii) predict fish responses to the loss of SCTLD-susceptible coral species. This three-stage approach helped us make informed predictions about the potential direct and indirect impacts of SCTLD on reef communities.

## RESULTS AND DISCUSSION

**Fig. 1.**
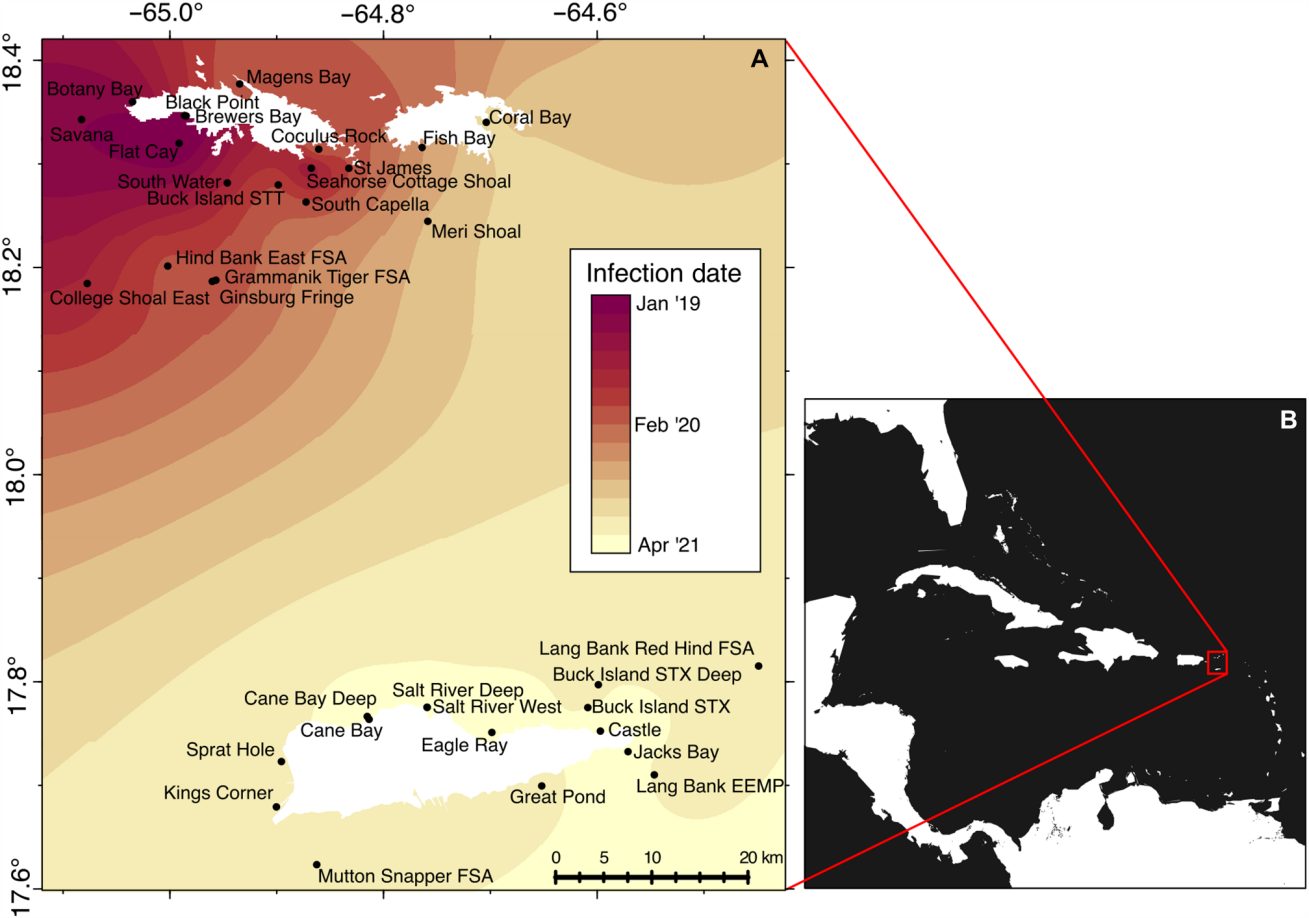
Stony coral tissue loss disease progressed throughout the U.S. Virgin Islands over a period of 27 months. Temporal progression of SCTLD through the 34 TCRMP sites represented by an interpolated heatmap (**A**) and inset showing study site locations within the broader Caribbean region (**B**). SCTLD was first documented in St. Thomas, and then spread through St. John, followed by St. Croix. In little more than 2 years, SCTLD had infected all 34 TCRMP sites in the USVI. This figure uses an ArcGIS base map with the following attribution: “Esri, TomTom, Garmin, FAO, NOAA, USGS, and EPA.”

SCTLD spread throughout the USVI over 27 months in a northwest-to-southeast direction (Fig. 1). This geographic trend is consistent with the disease spread across the broader Caribbean region (*50*). As previously reported in the Caribbean (*1*–*3*, *51*, *52*), our analyses of the TCRMP data indicate that, in the USVI, SCTLD was associated with declines in susceptible coral species that provide reef structure. Less expected was a decline in crustose coralline algae (CCA), which promotes coral persistence by cementing reefs together, facilitating coral recruitment (*53*), and suppressing macroalgae (*54*). It is not possible for us to determine whether the negative relationship between SCTLD and CCA is direct, caused by the pathogen infecting CCA, or indirect, mediated by the declines in susceptible coral or increases in other soft-bodied organisms. By reducing both hard coral and CCA, SCTLD threatens a reef’s bricks and mortar, respectively.

**Fig. 2.**
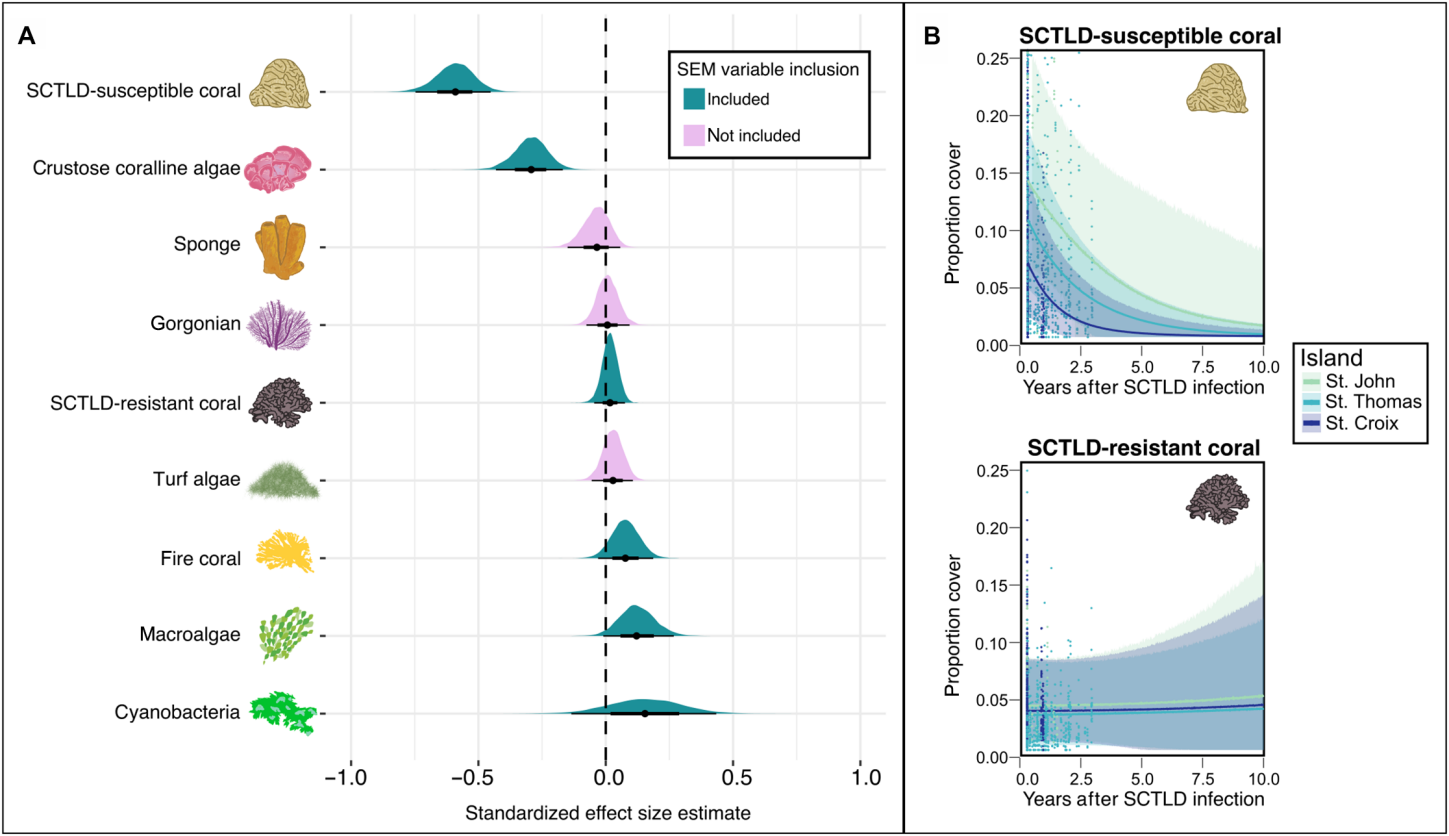
Stony coral tissue loss disease altered benthic communities in the U.S. Virgin Islands. Results from multilevel Bayesian regression models of benthic organism percent cover over time since initial SCTLD infection, based on TCRMP data. (**A**) Standardized effect size estimates with 95% credible intervals (CIs) are shown in thick black lines, and 65% CIs are shown in thin black lines of each stat-eye plot. Plots where CIs cross zero, shown with a vertical dashed black line, did not respond to SCTLD, whereas the further the CI is from zero, the stronger the response to SCTLD. (**B**) Ten-year predictions of absolute cover of SCTLD-susceptible and SCTLD-resistant species. Points represent real observations in the TCRMP data, whereas lines represent mean predicted values for each island in the TCRMP dataset, and shaded regions show 95% confidence intervals of these predictions.

The loss of SCTLD-susceptible coral (Fig. 2A) seemed to benefit competitive weedy species, as evidenced by increases in fire coral, macroalgae, and cyanobacteria (Fig. 2A); taxa associated with declining reefs (*55*), other marine diseases (*56*), and historical ecosystem collapse (*57*). After 10 years, the models projected that SCTLD-susceptible coral will have decreased on average by 97.6 ± 0.7% (Fig. 2B). Although SCTLD-resistant coral is predicted to increase on average by 46.4 ± 6.4%, declines in susceptible corals have not yet been offset by increases in resistant corals (Fig. 2B).

**Fig. 3.**
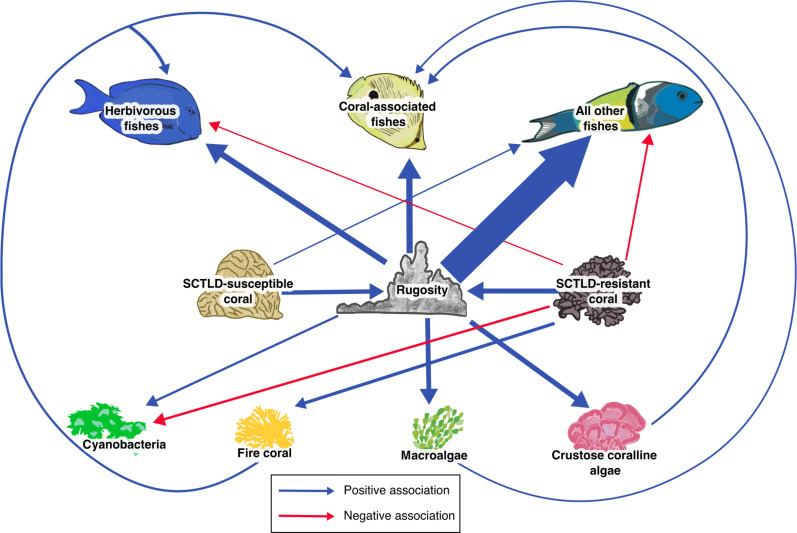
Existing networks between benthic organisms, rugosity, and fish are expected to influence SCTLD’s impact on Caribbean coral reefs. Path diagram showing all statistically significant relationships identified with the SEM of NCRMP data. Region, year, and depth are not shown but were included as random variables. Path weights are proportional to the mean effect sizes of the group-centered predictor variables. All benthic space holders (SCTLD-resistant coral, SCTLD-susceptible coral, cyanobacteria, fire coral, macroalgae, and crustose coralline algae) were included as predictors for all other variables. Rugosity was included as a predictor of the sessile benthic organisms and all three fish variables. Significant positive relationships are represented by blue arrows, insignificant relationships are not shown, and negative relationships are shown in red.

A Bayesian SEM of survey data from throughout the Caribbean region revealed that reef fish were directly and positively associated with rugosity, cover of SCTLD-susceptible coral, and cover of sessile benthic organisms (fire coral, macroalgae, and CCA) (Fig. 3 and fig. S1). Rugosity was the most important driver in our model, directly and positively linked to nearly every organism in the SEM, with greater effect sizes than other drivers in the system (Fig. 3 and fig. S1). The most likely biological explanation for these large effect sizes is that increased rugosity is associated with more available habitat and shelter. On coral reefs, rugosity is created primarily by stony corals, which grow in complex, irregular shapes, providing hiding places for mobile organisms and substrate for settlement and growth of sessile organisms (*34*, *58*). On average, SCTLD-susceptible and SCTLD-resistant coral contributed equally to rugosity (Fig. 3 and fig. S1).

**Fig. 4.**
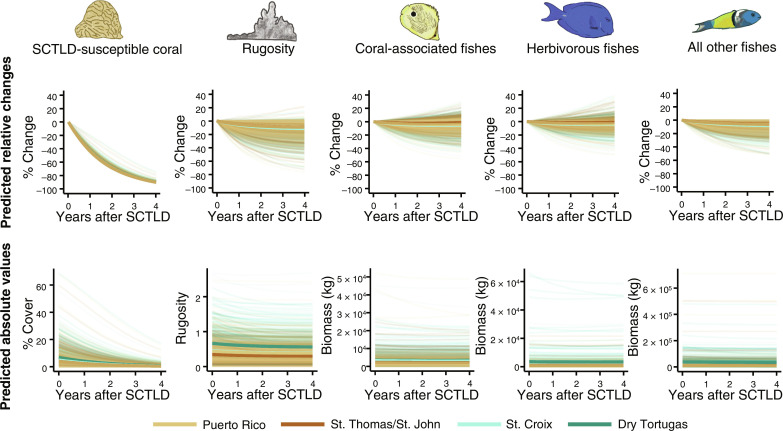
Following the introduction of SCTLD, reef fish responses are predicted to vary across functional groups. Predictions of absolute and relative changes after the introduction of SCTLD. Each thin line represents predictions at a site from the NCRMP dataset, and thick lines show regional mean values. Predictions were generated by applying rates of change in benthic cover associated with SCTLD observed in the TCRMP dataset to initial conditions in the NCRMP dataset, given relationships between variables that were quantified by the SEM. Predictions were generated out to 4 years after the initial SCTLD infection.

**Fig. 5.**
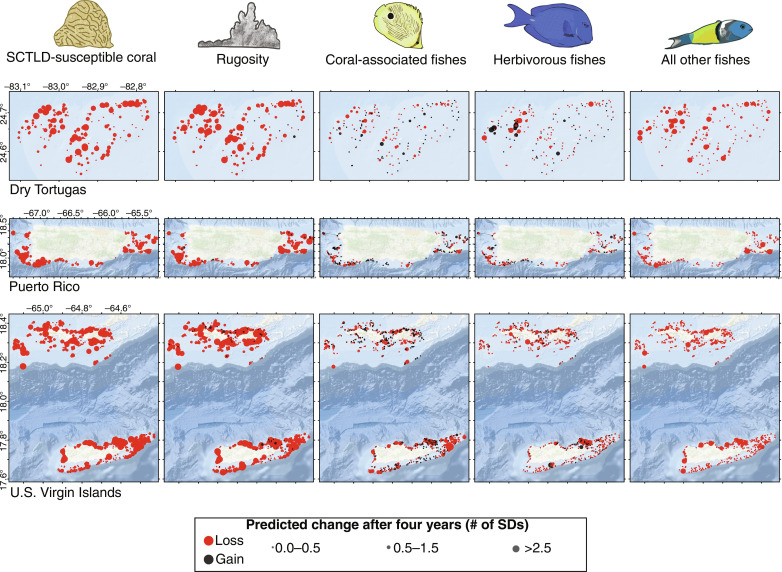
Community impacts of SCTLD are predicted to be highly geographically variable. Map of predicted changes to SCTLD-susceptible coral, rugosity, coral-associated fishes, herbivorous fishes, and all other fishes after 4 years of SCTLD presence. Point size represents the magnitude of predicted change after 4 years, expressed as the number of SDs from the initial values. Larger points represent the greatest predicted changes, red points represent sites where decreases are predicted, and black points represent sites with predicted increases. Latitude and longitude lines for each region (Dry Tortugas, Puerto Rico, and USVI) are shown in the leftmost map of each region. This figure uses an ArcGIS base map with the following attribution: “Esri, HERE, Garmin, GEBCO, EPA, FAO, FDEP, Foursquare, Kadaster Netherlands, METI/NASA, NaturalVue, NPS, SafeGraph, USVI GIS Division, USDA, and USGS.”

Fish communities are not predicted to collapse in response to SCTLD (Figs. 3 to 5). Fish had complex responses to variability in reef composition associated with the disease. Herbivorous fishes were positively associated with fire coral (Fig. 3 and fig. S1), which can provide shelter (*59*) and benefit indirectly from SCTLD (Fig. 2A). Coral-associated fish benefitted from macroalgae, fire coral, and CCA, but unexpectedly had no direct relationships with live scleractinian coral (Fig. 3 and fig. S1). Because macroalgae and fire coral are expected to benefit from SCTLD, and CCA is expected to decline (Fig. 2A), coral-associated fish will likely have a range of responses to SCTLD. Moreover, these results suggest that the rugosity of coral, rather than its status as alive or dead, per se, may underlie many coral-fish associations. “All other fishes” benefitted from the presence of SCTLD-susceptible coral but were negatively associated with SCTLD-resistant coral (Fig. 3 and fig. S1). This latter association suggests that predatory and non-coral associated fish may unexpectedly be the groups most indirectly affected by SCTLD. Thus, we expect that SCTLD will alter fish communities in complex ways associated with the initial proportion of susceptible corals on a reef and the extent to which their loss will reduce rugosity (Figs. 4 and 5).

Model simulations informed by both the modeled TCRMP data and relationships from the SEM predicted, on average, a 10% decline in rugosity in the 4 years following the introduction of SCTLD at a given site (Fig. 4). This is consistent with other observations of erosion following coral mortality (*60*–*63*). Because predictions were based on existing relationships between live coral and rugosity in the Caribbean, these modeled reductions in rugosity following SCTLD are likely underestimates, as bioerosion will continue to lower rugosity, even without additional coral death. On the other hand, compensatory growth of SCTLD-resistant coral could help restore rugosity over the long term.

Predicted responses of reef fish to the onset of SCTLD were variable. Although most fish communities were projected to change little, those at some sites were expected to decline by up to 50%, with the “other fish” category most sensitive to the coral disease (Figs. 4 and 5). This finding is consistent with a recent study demonstrating that live corals have had a surprisingly minor influence on the evolution of most coral reef fish (*64*). Butterflyfishes, which are facultative corallivores in this region, have even been found to prefer SCTLD-infected coral (*65*), which may underlie some of the positive associations between coral-associated fish and the spread of SCTLD. Although SCTLD was projected to decrease reef rugosity, this effect was likely buffered by the positive associations between fish and the compensatory growth of soft-bodied organisms projected to benefit from SCTLD (Fig. 2A).

The loss of SCTLD-susceptible corals is expected on any reefs reached by the disease, with the magnitude of coral loss driven primarily by the initial abundance of those corals. Rugosity is expected to mostly decline moderately, though it might increase in a few areas with the growth of species that are either not affected or indirectly benefit from SCTLD. Consequently, changes to herbivorous and coral-associated fishes are expected to be highly variable across the region, with neighboring reefs often having opposite responses. However, changes to these two fish guilds will often be in parallel at a given site, whereas all other fishes consistently decline, to various magnitudes, in response to SCTLD (Fig. 5).

Years of detailed monitoring data from the Caribbean allowed us to analyze the potential for SCTLD to affect coral reef communities beyond killing infected corals. These effects were complex, with winners and losers that could accelerate the transition of reefs to dominance by nuisance benthic species with altered fish communities. As SCTLD continues to progress beyond its current range in the Caribbean, we expect variable and indirect changes to associated benthic and fish communities that rely on coral for habitat and other resources, primarily through the extent that coral death and subsequent erosion will reduce rugosity. Some sites will be heavily affected, others will see little change, and yet others will see increases in some fishes. Continued monitoring in the USVI and other regions will reveal whether these trends continue with time or reach alternative equilibria. Manipulative studies could help reveal the mechanisms behind the predicted changes in associated components of the community. Having distinguished the features of Caribbean coral reefs that predict community vulnerability to SCTLD, we can now identify which reefs are most at risk of ecosystem-level impacts of disease and how they are likely to change. Highly affected reefs, or those at risk of future impacts, might benefit most from restoration with resistant coral species.

## MATERIALS AND METHODS

We used detailed reef community data collected throughout the SCTLD epidemic by the TCRMP in the USVI and multilevel Bayesian models to assess the impacts of SCTLD on benthic communities. Then, we built a Bayesian SEM with data from the NCRMP, spanning coral reefs in the USVI, Puerto Rico, and the Dry Tortugas, to analyze existing relationships between (i) the benthic functional groups found to respond to SCTLD in the first part of our analysis and (ii) the biomass of coral-associated, herbivorous, and all other fishes. Benthic organisms that responded to SCTLD in the USVI (Fig. 2) were included in the SEM, as well as reef rugosity and three groups of fishes—coral-associated fishes, herbivorous fishes, and all other reef fishes. Last, we used the results from the TCRMP models and the SEM to generate predictions of reef fish composition following the introduction of SCTLD. Data collection is summarized below with details available from the cited data providers. All analyses were performed using R statistical software (*66*).

### TCRMP benthic data and processing

The TCRMP benthic survey data used in this study were collected at 34 sites across St. John, St. Thomas, and St. Croix in the USVI from 2017 to 2021 (*48*). Fixed benthic video transects were processed according to a standardized protocol (*67*), resulting in species or genus-level benthic composition data. Since SCTLD arrived in the USVI, the TCRMP surveyors have tracked its progression at their sites. Corals at each site were monitored for evidence of SCTLD, and resulting site-specific dates of first infection are included in the data (Fig. 1).

Before conducting statistical analyses on species-level coral and benthic data from the TCRMP dataset, we calculated the percent cover of benthic species that we assigned to nine distinct functional groups—four algal types: CCA, cyanobacteria, macroalgae, and turf algae; two scleractinian coral types: SCTLD-susceptible and SCTLD-resistant coral; fire coral; gorgonians; and sponges. Zoanthids were omitted because of their rarity at the depths surveyed. This categorization reduced resolution but made our analyses tractable and minimized errors associated with small sample sizes and other uncertainties at the species level. We categorized corals as “SCTLD-susceptible” and “SCTLD-resistant” according to published reports (*51*, *68*) (table S1).

### Multilevel Bayesian models of SCTLD impact on benthic communities in USVI

The impact of SCTLD on benthic communities in the USVI was assessed using multilevel Bayesian models. The effect of SCTLD was represented in each model by a “years since infection” variable indicating the amount of time elapsed between when SCTLD was first observed at the site and when a given survey was conducted. Surveys conducted before SCTLD arrived were assigned 0’s in the “years since infection” variable. We ran a separate multilevel Bayesian model for each benthic functional group using the zero-inflated beta distribution and the formula “% cover ~ 1 + yearsSinceInfection + (1 + yearsSinceInfection|Site).”

The “phi” and “zi” terms for the spread and zero inflation of the response, respectively, were modeled with the same predictors. The effect of SCTLD was allowed to vary by site with the inclusion of the random slope term (1 + yearsSinceInfection|Site). The random slope terms allowed for variation in the responses of benthic space holders to SCTLD at different sites while still estimating an overall effect size. The “years since infection” variable was first modeled using a smoothing term to allow for nonlinear effects of SCTLD on benthic communities. However, smoothing terms did not improve models and were therefore removed. Intercept parameter priors were chosen on the basis of group means. Uninformative, weakly regularizing priors were used for slope parameters, which are used to estimate effect sizes and to reduce the likelihood of overfitting (*69*). SD parameter priors were modeled using the exponential distribution with the assumption that, although large variances are possible, they are less likely than small variances.

Model performance was assessed using rhat values, effective sample sizes, posterior predictive checks, Watanabe-Akaike information criterion (WAIC) scores, and leave-one-out (LOO) cross-validation scores. On the basis of these criteria, we kept the most defensible models. Standardized estimated effect sizes, plots of posterior distributions, and 95% credible intervals were used to assess the strength and direction of relationships between the benthic functional groups and SCTLD. We used the predict() function from the R *stats* package to generate predictions about changes in the percent cover of susceptible and resistant coral species over a 10-year period.

A key assumption of the TCRMP models is that changes in the percent cover of benthic space holders over time were primarily due to either direct or indirect effects of SCTLD on community dynamics and that no other major changes to coral reef community dynamics exhibited a similar timing and pattern of spread across the USVI as SCTLD. Because the spread of SCTLD over this period is documented with high temporal resolution (each site was visited quarterly), and the estimated effects of SCTLD on other benthic space holders are biologically reasonable, we are confident in this assumption.

### NCRMP benthic and fish community monitoring and data processing

The NCRMP coral demography survey and benthic community data used in this study were collected in situ along 10 m × 1 m transects, with site locations selected using a random sampling design stratified by regions, reef zones, and depth. In each coral demography transect, the size and quantity of any coral greater than 5 cm in diameter were measured. Within the transect area, for every colony with live tissue, the total colony size was estimated by measuring the colony’s maximum diameter and longest perpendicular diameter. At each site, divers recorded information about the physical habitat (i.e., vertical relief, depth, and bottom type) and measured the maximum vertical relief at each meter of the transect to calculate rugosity. At each site, benthic composition was surveyed using line-point intercept methods, identifying species every 15 cm along a 15-m transect, for a total of 100 points per site (*70*).

Fish community surveys were conducted at the same sites as benthic transects, according to standard NCRMP Reef Visual Census (RVC) protocols (*70*). Briefly, two divers each conducted a 7.5-m radius, 15-min point count fish survey. Once the cylinders were established, divers used 5 min to identify all species in the cylinder. In the remaining 10 min, the length and number of fish were recorded. The addition of species was permitted after the first 5 min, but divers recorded when each additional species was found.

As with the TCRMP data, we grouped NCRMP benthic taxa into functional groups before analysis. Total areas of live SCTLD-susceptible and SCTLD-resistant coral were calculated from coral demography data. Proportional areas of the benthic functional groups that responded to SCTLD in the TCRMP analysis were calculated from the benthic line-point intercept data. Benthic data were filtered to remove surveys where rugosity was not measured. To account for spatial autocorrelation at the regional scale and to minimize the influence of missing variables, we used group-centered predictors. Group-centering involves calculating averages and SDs from subsets of the data with common characteristics (in this case, data from the same subregion) and applying the following formula: group-centered value = (site value − subregional mean)/subregional SD. Thus, the group-centered value reflects how different a site is from other, nearby sites, and consequently provides a more easily interpretable estimate of the relationship between two variables that minimizes the influence of external drivers (*71*). By including these values, we ensured that models were analyzing variation within and not across regions, which may be driven by more complex biogeographic relationships.

NCRMP fish survey data were also processed for use in the SEM. For each RVC survey, we calculated the total biomass for subsets of the fish community—coral-associated fishes, herbivorous fishes, and all other fishes. Biomass estimates of individual fish were calculated using length estimates from the dataset and length-weight relationships published on FishBase (*72*). Fish were categorized into groups (coral-associated fishes, herbivorous fishes, and all other fishes) that we hypothesized would respond differently to changes in benthic communities linked to SCTLD. First, we used the global database of fish with known associations with coral from Strona *et al*. (*40*) and grouped obligate corallivores, facultative corallivores, and fish known to use live coral for habitat into a category called “coral-associated fish” (*73*–*75*). Herbivorous, non-coral-associated fish were identified by pairing species in the NCRMP dataset with FishBase data on diets and trophic groups. The remaining fish were categorized into the “all other fish” group, which includes all non-coral-associated, non-herbivorous reef fish (dataset S1).

NCRMP fish and benthic surveys were not always conducted together, so data processing entailed a step to ensure spatial and temporal proximity of benthic and fish surveys. Surveys considered paired were conducted within 100 m and 90 days of each other, with the assumption that these ranges were small enough that benthic and fish communities within these ranges were “associated” with each other. Following this spatial and temporal matching and data filtering, 1610 paired fish and benthic surveys, from 2016 to 2021, remained for use in subsequent analyses.

### Structural equation model of biotic communities and physical reef structure

After using the TCRMP dataset to determine which benthic organisms were most affected by SCTLD, we used an SEM to assess the relationships of these benthic taxa with reef fish communities. We first used directed acyclic graphs to map our hypotheses about benthic communities, fish communities, and physical reef traits onto a path diagram (*76*). Pairwise correlations between variables were examined graphically before model building to inform decisions about random variable inclusion and shape parameters. We then used the package *brms* (version 2.18.0) in R statistical software (version 4.2.1) to build an SEM testing these hypotheses.

SEMs can be powerful tools for distinguishing the relative contributions of factors within a system but require prior assumptions about the directionality of relationships to be tested. Relationships in an SEM must be encoded as a single, direct, and directional pathway, which is a simplification made for the sake of model tractability. In reality, the relationships modeled (such as those between benthic cover and fish biomass) likely consist of many pathways, direct and indirect, that flow in both directions; the modeled association reflects the net sum of these pathways. Proportional cover of SCTLD-susceptible and SCTLD-resistant coral was included as true exogenous variables and predictors of rugosity and the benthic functional groups included in the model (CCA, macroalgae, fire coral, and cyanobacteria). Rugosity was included in the model because of known positive relationships between fish and structural complexity. By including paths both (i) from coral area to rugosity to fish and (ii) from coral area directly to fish, we designed the model to determine whether live SCTLD-susceptible and SCTLD-resistant coral uniquely contribute to fish communities apart from their contributions to rugosity. Benthic functional groups were used to predict fish biomass because these may structure fish communities through trophic and non-trophic interactions (*77*–*79*). Although many other model structures would have been possible, we based our model on assumptions informed by data, literature, and our familiarity with Caribbean coral reef systems.

Before building the complete SEM, we first built a multilevel Bayesian model for each individual response variable to ensure proper parameters and distributional families were selected. Rather than including raw values for each predictor variable, group-centered means and regional means of each predictor variable were used to minimize the potential influence of missing variables (*71*). Distributions were specified for each response according to the processes that generated the data. If multiple distributions were candidates to describe a given response variable, we built models using each distribution and subsequently tested the fit of each model. In all cases, posterior predictive checks, WAIC scores, and LOO weights clearly identified the distribution that best described the data, and this distribution was used in the final model. Priors were chosen using the same principles we used to select priors for the Bayesian regressions of TCRMP data. A prior sensitivity analysis showed our selected priors did not unduly influence effect size estimates (fig. S2). After building individual models, the SEM was compiled using the full set of hypothesized predictors. Then, any predictors for which the 95% credible interval of the effect estimate overlapped with zero were removed from the model. The reduced models were compared to the full model using the model_weights() function based on WAIC and LOO scores. We used posterior predictive checks on reduced models to confirm that the models’ posterior distributions matched the real data for each individual response. Estimated sample size and rhat values were used to confirm model convergence. Standardized effect size estimates were calculated from the posterior distributions and were used to assess the strength, direction, and significance of relationships.

### Predicting post-SCTLD reef fish communities

We used the posterior estimates from the TCRMP models and results from the SEM to predict the consequences of plausible SCTLD coral loss scenarios on reef fish communities. Two hundred eight posterior draws, each representing 1 week after SCTLD infection, were simulated for each response variable to predict how reef communities would respond to SCTLD outbreaks. For each functional group, we plotted absolute and relative changes over time since SCTLD was first introduced (Fig. 4). Last, we mapped the cumulative predicted changes (in % cover) spatially to visualize where impacts are likely to be most severe (Fig. 5).

## Supplementary Materials

### This PDF file includes:

Figs. S1 and S2 Table S1 References

## References

[1] L. Alvarez-Filip, F. J. González-Barrios, E. Pérez-Cervantes, A. Molina-Hernández, N. Estrada-Saldívar, Stony coral tissue loss disease decimated Caribbean coral populations and reshaped reef functionality. Commun. Biol. 5, 440 (2022). 35681037 10.1038/s42003-022-03398-6 PMC9184636

[2] M. E. Brandt, R. S. Ennis, S. S. Meiling, J. Townsend, K. Cobleigh, A. Glahn, J. Quetel, V. Brandtneris, L. M. Henderson, T. B. Smith, The emergence and initial impact of Stony Coral Tissue Loss Disease (SCTLD) in the United States Virgin Islands. Front. Mar. Sci. 8, 715329 (2021).

[3] C. J. Walton, N. K. Hayes, D. S. Gilliam, Impacts of a regional, multi-year, multi-species coral disease outbreak in Southeast Florida. Front. Mar. Sci. 5, 323 (2018).

[4] W. F. Precht, B. E. Gintert, M. L. Robbart, R. Fura, R. van Woesik, Unprecedented disease-related coral mortality in Southeastern Florida. Sci. Rep. 6, 31374 (2016). 27506875 10.1038/srep31374 PMC4979204

[5] J. L. Meyer, J. Castellanos-Gell, G. S. Aeby, C. C. Häse, B. Ushijima, V. J. Paul, Microbial community shifts associated with the ongoing Stony Coral Tissue Loss Disease Outbreak on the Florida Reef Tract. Front. Microbiol. 10, 2244 (2019). 31608047 10.3389/fmicb.2019.02244 PMC6769089

[6] C. C. Becker, M. Brandt, C. A. Miller, A. Apprill, Microbial bioindicators of Stony Coral Tissue Loss Disease identified in corals and overlying waters using a rapid field-based sequencing approach. Environ. Microbiol. 24, 1166–1182 (2022). 34431191 10.1111/1462-2920.15718

[7] J. Meyer, Development of probiotics and alternative treatments for stony coral tissue loss disease (Florida Department of Environmental Protection, 2021).

[8] K. L. Neely, K. A. Macaulay, E. K. Hower, M. A. Dobler, Effectiveness of topical antibiotics in treating corals affected by Stony Coral Tissue Loss Disease. PeerJ 8, e9289 (2020). 32551200 10.7717/peerj.9289 PMC7292019

[9] B. Ushijima, S. P. Gunasekera, J. L. Meyer, J. Tittl, K. A. Pitts, S. Thompson, J. M. Sneed, Y. Ding, M. Chen, L. Jay Houk, G. S. Aeby, C. C. Häse, V. J. Paul, Chemical and genomic characterization of a potential probiotic treatment for stony coral tissue loss disease. Commun. Biol. 6, 248 (2023). 37024599 10.1038/s42003-023-04590-y PMC10079959

[10] K. M. Beavers, E. W. Van Buren, A. M. Rossin, M. A. Emery, A. J. Veglia, C. E. Karrick, N. J. MacKnight, B. A. Dimos, S. S. Meiling, T. B. Smith, A. Apprill, E. M. Muller, D. M. Holstein, A. M. S. Correa, M. E. Brandt, L. D. Mydlarz, Stony coral tissue loss disease induces transcriptional signatures of in situ degradation of dysfunctional Symbiodiniaceae. Nat. Commun. 14, 2915 (2023). 37217477 10.1038/s41467-023-38612-4 PMC10202950

[11] T. M. Work, T. M. Weatherby, J. H. Landsberg, Y. Kiryu, S. M. Cook, E. C. Peters, Viral-like particles are associated with endosymbiont pathology in Florida corals affected by Stony Coral Tissue Loss Disease. Front. Mar. Sci. 8, 750658 (2021).

[12] K. L. Neely, C. L. Lewis, K. S. Lunz, L. Kabay, Rapid population decline of the pillar coral *Dendrogyra cylindrus* along the Florida Reef Tract. Front. Mar. Sci. 8, 656515 (2021).

[13] N. K. Hayes, C. J. Walton, D. S. Gilliam, Tissue loss disease outbreak significantly alters the Southeast Florida stony coral assemblage. Front. Mar. Sci. 9, 975894 (2022).

[14] I. Hewson, J. B. Button, B. M. Gudenkauf, B. Miner, A. L. Newton, J. K. Gaydos, J. Wynne, C. L. Groves, G. Hendler, M. Murray, S. Fradkin, M. Breitbart, E. Fahsbender, K. D. Lafferty, A. M. Kilpatrick, C. M. Miner, P. Raimondi, L. Lahner, C. S. Friedman, S. Daniels, M. Haulena, J. Marliave, C. A. Burge, M. E. Eisenlord, C. D. Harvell, Densovirus associated with sea-star wasting disease and mass mortality. Proc. Natl. Acad. Sci. U.S.A. 111, 17278–17283 (2014). 25404293 10.1073/pnas.1416625111 PMC4260605

[15] C. J. Feehan, R. E. Scheibling, Effects of sea urchin disease on coastal marine ecosystems. Mar. Biol. 161, 1467–1485 (2014).

[16] H. A. Lessios, Mass mortality of *Diadema antillarum* in the Caribbean: What have we learned? Annu. Rev. Ecol. Syst. 19, 371–393 (1988).

[17] K. D. Lafferty, C. D. Harvell, The role of infectious diseases in marine communities, in *Marine Community Ecology and Conservation* (Sinauer Associates Inc., 2014), pp. 85–108.

[18] M. Behrens, K. Lafferty, Effects of marine reserves and urchin disease on southern Californian rocky reef communities. Mar. Ecol. Prog. Ser. 279, 129–139 (2004).

[19] M. Reaka-Kudla, The global biodiversity of coral reefs: A comparison with rainforests, in *Biodiversity II: Understanding and Protecting Our Natural Resources* (Joseph Henry/National Academy Press, 1997), pp. 83–108.

[20] J. B. C. Jackson, M. X. Kirby, W. H. Berger, K. A. Bjorndal, L. W. Botsford, B. J. Bourque, R. H. Bradbury, R. Cooke, J. Erlandson, J. A. Estes, T. P. Hughes, S. Kidwell, C. B. Lange, H. S. Lenihan, J. M. Pandolfi, C. H. Peterson, R. S. Steneck, M. J. Tegner, R. R. Warner, Historical overfishing and the recent collapse of coastal ecosystems. Science 293, 629–637 (2001). 11474098 10.1126/science.1059199

[21] J. B. C. Jackson, Reefs since Columbus. Coral Reefs 16, S23–S32 (1997).

[22] W. D. Liddell, S. L. Ohlhorst, Changes in benthic community composition following the mass mortality of *Diadema* at Jamaica. J. Exp. Mar. Biol. Ecol. 95, 271–278 (1986).

[23] R. C. Carpenter, Mass mortality of *Diadema antillarum*. Mar. Biol. 104, 67–77 (1990).

[24] L. Cheng, K. von Schuckmann, J. P. Abraham, K. E. Trenberth, M. E. Mann, L. Zanna, M. H. England, J. D. Zika, J. T. Fasullo, Y. Yu, Y. Pan, J. Zhu, E. R. Newsom, B. Bronselaer, X. Lin, Past and future ocean warming. Nat. Rev. Earth Environ. 3, 776–794 (2022).

[25] A. C. Baker, P. W. Glynn, B. Riegl, Climate change and coral reef bleaching: An ecological assessment of long-term impacts, recovery trends and future outlook. Estuar. Coast. Shelf Sci. 80, 435–471 (2008).

[26] V. Schoepf, A. G. Grottoli, S. J. Levas, M. D. Aschaffenburg, J. H. Baumann, Y. Matsui, M. E. Warner, Annual coral bleaching and the long-term recovery capacity of coral. Proc. Biol. Sci. 282, 20151887 (2015). 26582020 10.1098/rspb.2015.1887 PMC4685810

[27] R. B. Aronson, W. F. Precht, White-band disease and the changing face of Caribbean coral reefs. Hydrobiologia 460, 25–38 (2001).

[28] K. G. Kuta, L. L. Richardson, Abundance and distribution of black band disease on coral reefs in the northern Florida Keys. Coral Reefs 15, 219–223 (1996).

[29] A. W. Bruckner, R. J. Bruckner, Consequences of yellow band disease (YBD) on *Montastraea annularis* (species complex) populations on remote reefs off Mona Island, Puerto Rico. Dis. Aquat. Organ. 69, 67–73 (2006). 16703767 10.3354/dao069067

[30] K. D. Lafferty, J. W. Porter, S. E. Ford, Are diseases increasing in the ocean? Annu. Rev. Ecol. Evol. Syst. 35, 31–54 (2004).

[31] K. E. Kovalenko, S. M. Thomaz, D. M. Warfe, Habitat complexity: Approaches and future directions. Hydrobiologia 685, 1–17 (2012).

[32] D. J. Coker, S. K. Wilson, M. S. Pratchett, Importance of live coral habitat for reef fishes. Rev. Fish Biol. Fish. 24, 89–126 (2014).

[33] E. S. Darling, N. A. J. Graham, F. A. Januchowski-Hartley, K. L. Nash, M. S. Pratchett, S. K. Wilson, Relationships between structural complexity, coral traits, and reef fish assemblages. Coral Reefs 36, 561–575 (2017).

[34] A. Rogers, J. L. Blanchard, S. P. Newman, C. S. Dryden, P. J. Mumby, High refuge availability on coral reefs increases the vulnerability of reef-associated predators to overexploitation. Ecology 99, 450–463 (2018). 29328509 10.1002/ecy.2103

[35] A. R. Harborne, A. Rogers, Y.-M. Bozec, P. J. Mumby, Multiple stressors and the functioning of coral reefs. Ann. Rev. Mar. Sci. 9, 445–468 (2017). 10.1146/annurev-marine-010816-060551 27575738

[36] M. A. Hixon, J. P. Beets, Predation, prey refuges, and the structure of coral-reef fish assemblages. Ecol. Monogr. 63, 77–101 (1993).

[37] S. J. Brandl, M. J. Emslie, D. M. Ceccarelli, Z. T. Richards, Habitat degradation increases functional originality in highly diverse coral reef fish assemblages. Ecosphere 7, e01557 (2016).

[38] D. A. Feary, G. R. Almany, M. I. McCormick, G. P. Jones, Habitat choice, recruitment and the response of coral reef fishes to coral degradation. Oecologia 153, 727–737 (2007). 17566781 10.1007/s00442-007-0773-4

[39] G. R. Russ, J. R. Rizzari, R. A. Abesamis, A. C. Alcala, Coral cover a stronger driver of reef fish trophic biomass than fishing. Ecol. Appl. 31, e02224 (2021). 32866333 10.1002/eap.2224 PMC7816266

[40] G. Strona, K. D. Lafferty, S. Fattorini, P. S. A. Beck, F. Guilhaumon, R. Arrigoni, S. Montano, D. Seveso, P. Galli, S. Planes, V. Parravicini, Global tropical reef fish richness could decline by around half if corals are lost. Proc. Biol. Sci. 288, 20210274 (2021). 34187190 10.1098/rspb.2021.0274 PMC8242923

[41] M. L. Reaka-Kudla, J. S. Feingold, W. Glynn, Experimental studies of rapid bioerosion of coral reefs in the Galápagos Islands. Coral Reefs 15, 101–107 (1996).

[42] B. Gratwicke, M. R. Speight, The relationship between fish species richness, abundance and habitat complexity in a range of shallow tropical marine habitats. J. Fish Biol. 66, 650–667 (2005).

[43] B. Gratwicke, M. Speight, Effects of habitat complexity on Caribbean marine fish assemblages. Mar. Ecol. Prog. Ser. 292, 301–310 (2005).

[44] A. R. Harborne, P. J. Mumby, R. Ferrari, The effectiveness of different meso-scale rugosity metrics for predicting intra-habitat variation in coral-reef fish assemblages. Environ. Biol. Fishes 94, 431–442 (2012).

[45] C. I. Elliff, I. R. Silva, Coral reefs as the first line of defense: Shoreline protection in face of climate change. Mar. Environ. Res. 127, 148–154 (2017). 28366280 10.1016/j.marenvres.2017.03.007

[46] M. Spalding, L. Burke, S. A. Wood, J. Ashpole, J. Hutchison, P. zu Ermgassen, Mapping the global value and distribution of coral reef tourism. Mar. Policy 82, 104–113 (2017).

[47] H. Cesar, L. Burke, L. Pet-Soede, The economics of worldwide coral reef degradation (International Coral Reef Action Network, 2003).

[48] R. S. Ennis, S. L. Heidmann, L. M. Henderson, M. Warham, T. B. Smith, The United States Virgin Islands Territorial Coral Reef Monitoring Program (2021); https://sites.google.com/site/usvitcrmp/tcrmp-reports?authuser=0.

[49] NOAA National Centers for Environmental Information, *National Coral Reef Monitoring Program: Benthic, Coral Demography, and Reef Fish Visual Census data* (2021).

[50] AGRRA, Map of coral cover of susceptible coral species to SCTLD (2023); www.agrra.org.

[51] N. Estrada-Saldívar, B. A. Quiroga-García, E. Pérez-Cervantes, O. O. Rivera-Garibay, L. Alvarez-Filip, Effects of the Stony Coral Tissue Loss Disease outbreak on coral communities and the benthic composition of Cozumel reefs. Front. Mar. Sci. 8, 632777 (2021).

[52] S. S. Meiling, E. M. Muller, D. Lasseigne, A. Rossin, A. J. Veglia, N. MacKnight, B. Dimos, N. Huntley, A. M. S. Correa, T. B. Smith, D. M. Holstein, L. D. Mydlarz, A. Apprill, M. E. Brandt, Variable species responses to experimental stony coral tissue loss disease (SCTLD) exposure. Front. Mar. Sci. 8, 670829 (2021).

[53] J. M. Sneed, K. H. Sharp, K. B. Ritchie, V. J. Paul, The chemical cue tetrabromopyrrole from a biofilm bacterium induces settlement of multiple Caribbean corals. Proc. Biol. Sci. 281, 20133086 (2014). 24850918 10.1098/rspb.2013.3086 PMC4046396

[54] M. J. A. Vermeij, M. L. Dailer, C. M. Smith, Crustose coralline algae can suppress macroalgal growth and recruitment on Hawaiian coral reefs. Mar. Ecol. Prog. Ser. 422, 1–7 (2011).

[55] T. P. Hughes, Catastrophes, phase shifts, and large-scale degradation of a Caribbean coral reef. Science 265, 1547–1551 (1994). 17801530 10.1126/science.265.5178.1547

[56] J. L. Meyer, S. P. Gunasekera, R. M. Scott, V. J. Paul, M. Teplitski, Microbiome shifts and the inhibition of quorum sensing by Black Band Disease cyanobacteria. ISME J. 10, 1204–1216 (2016). 26495995 10.1038/ismej.2015.184 PMC5029210

[57] P. Copper, Ancient reef ecosystem expansion and collapse. Coral Reefs 13, 3–11 (1994).

[58] N. A. J. Graham, K. L. Nash, The importance of structural complexity in coral reef ecosystems. Coral Reefs 32, 315–326 (2013).

[59] I. C. S. Leal, M. E. de Araújo, S. R. da Cunha, P. H. C. Pereira, The influence of fire-coral colony size and agonistic behaviour of territorial damselfish on associated coral reef fish communities. Mar. Environ. Res. 108, 45–54 (2015). 25956544 10.1016/j.marenvres.2015.04.009

[60] G. Roff, J. Zhao, P. J. Mumby, Decadal-scale rates of reef erosion following El Niño-related mass coral mortality. Glob. Chang. Biol. 21, 4415–4424 (2015). 26113199 10.1111/gcb.13006

[61] J. Morais, R. Morais, S. B. Tebbett, D. R. Bellwood, On the fate of dead coral colonies. Funct. Ecol. 36, 3148–3160 (2022).

[62] A. Molina-Hernández, F. Medellín-Maldonado, I. D. Lange, C. T. Perry, L. Álvarez-Filip, Coral reef erosion: In situ measurement on different dead coral substrates on a Caribbean reef. Limnol. Oceanogr. 67, 2734–2749 (2022).

[63] Y.-M. Bozec, L. Alvarez-Filip, P. J. Mumby, The dynamics of architectural complexity on coral reefs under climate change. Glob. Chang. Biol. 21, 223–235 (2015). 25099220 10.1111/gcb.12698

[64] A. C. Siqueira, P. Muruga, D. R. Bellwood, On the evolution of fish–coral interactions. Ecol. Lett. 26, 1348–1358 (2023). 37222494 10.1111/ele.14245

[65] K. R. Noonan, M. J. Childress, Association of butterflyfishes and stony coral tissue loss disease in the Florida Keys. Coral Reefs 39, 1581–1590 (2020).

[66] R Core Team, *R: A Language and Environment for Statistical Computing, version 4.2.2* (R Foundation for Statistical Computing, 2022); www.R-project.org/.

[67] R. S. Ennis, S. L. Heidmann, L. M. Henderson, M. Warham, T. B. Smith, The United States Virgin Islands Territorial Coral Reef Monitoring Program (2021); https://sites.google.com/site/usvitcrmp/tcrmp-reports?authuser=0.

[68] M. E. Brandt, Stony Coral Tissue Loss Disease (SCTLD) case definition (Florida Department of Environmental Protection, 2018); https://floridadep.gov/rcp/coral/documents/stony-coral-tissue-loss-disease-sctld-case-definition.

[69] R. McElreath, *Statistical Rethinking: A Bayesian Course with Examples in R and Stan* (CRC Press/Taylor & Francis Group, 2016), Chapman & Hall/CRC Texts in Statistical Science Series.

[70] NOAA National Centers for Environmental Information, *Coral Reef Conservation Program Documentation for NOAA’s Coral Reef Conservation Program (CRCP) National Coral Reef Monitoring Program (NCRMP) data archived at NCEI*. Dataset, NCEI Accession 0157633 (2016); www.ncei.noaa.gov/archive/accession/0157633.

[71] Y. Mundlak, On the pooling of time series and cross section data. Econometrica 46, 69–85 (1978).

[72] C. Boettiger, D. T. Lang, P. C. Wainwright, rfishbase: Exploring, manipulating and visualizing FishBase data from R. J. Fish Biol. 81, 2030–2039 (2012). 23130696 10.1111/j.1095-8649.2012.03464.x

[73] A. J. Cole, M. S. Pratchett, G. P. Jones, Diversity and functional importance of coral-feeding fishes on tropical coral reefs. Fish Fish. 9, 286–307 (2008).

[74] K. L. Nash, N. A. J. Graham, S. Jennings, S. K. Wilson, D. R. Bellwood, Herbivore cross-scale redundancy supports response diversity and promotes coral reef resilience. J. Appl. Ecol. 53, 646–655 (2016).

[75] N. A. J. Graham, P. Chabanet, R. D. Evans, S. Jennings, Y. Letourneur, M. Aaron MacNeil, T. R. McClanahan, M. C. Öhman, N. V. C. Polunin, S. K. Wilson, Extinction vulnerability of coral reef fishes. Ecol. Lett. 14, 341–348 (2011). 21320260 10.1111/j.1461-0248.2011.01592.x PMC3627313

[76] K. Thulasiraman, M. N. S. Swamy, 5.7 acyclic directed graphs, in *Graphs: Theory and Algorithms* (Wiley, 1992), pp. 118–119.

[77] C. E. L. Ferreira, J. E. A. Goncalves, R. Coutinho, Community structure of fishes and habitat complexity on a tropical rocky shore. Environ. Biol. Fishes 61, 353–369 (2001).

[78] A. M. Canterle, L. T. Nunes, L. Fontoura, H. A. Maia, S. R. Floeter, Reef microhabitats mediate fish feeding intensity and agonistic interactions at Príncipe Island Biosphere Reserve, Tropical Eastern Atlantic. Mar. Ecol. 41, e12609 (2020).

[79] K. M. Chong-Seng, T. D. Mannering, M. S. Pratchett, D. R. Bellwood, N. A. J. Graham, The influence of coral reef benthic condition on associated fish assemblages. PLOS ONE 7, e42167 (2012). 22870294 10.1371/journal.pone.0042167 PMC3411644

[80] National Centers for Coastal Ocean Science (NCCOS), NOAA National Centers for Environmental Information, *National Coral Reef Monitoring Program: Assessment of Coral Reef Benthic Communities in the U.S. Virgin Islands* (2016); 10.7289/V5WW7FQK.

[81] National Centers for Coastal Ocean Science (NCCOS), Southeast Fisheries Science Center (SEFSC), NOAA National Centers for Environmental Information, *National Coral Reef Monitoring Program: Assessment of coral reef benthic communities in Puerto Rico* (2018); 10.7289/V5PG1Q23.

[82] NOAA Southeast Fisheries Science Center, NOAA National Centers for Coastal Ocean Science, NOAA National Centers for Environmental Information, *National Coral Reef Monitoring Program: Assessment of Coral Reef Benthic Communities in the Florida Reef Tract* (2018); 10.7289/v5xw4h4z.

[83] National Centers for Coastal Ocean Science (NCCOS), NOAA National Centers for Environmental Information, *National Coral Reef Monitoring Program: Assessment of Coral Reef Fish Communities in the U.S. Virgin Islands* (2016); 10.7289/V5F769MM.

[84] National Centers for Coastal Ocean Science (NCCOS), Southeast Fisheries Science Center (SEFSC), NOAA National Centers for Environmental Information, *National Coral Reef Monitoring Program: Assessment of Coral Reef Fish Communities in Puerto Rico* (2018); 10.7289/V5T72FRZ.

[85] NOAA Southeast Fisheries Science Center, NOAA National Centers for Coastal Ocean Science, NOAA National Centers for Environmental Information, *National Coral Reef Monitoring Program: Assessment of Coral Reef Fish Communities in the Florida Reef Tract* (2018); 10.7289/v52n50ks.

[86] WoRMS Editorial Board (2024). World Register of Marine Species, https://www.marinespecies.org at VLIZ [accessed 23 March 2024] doi:10.14284/170.

