# Supplementary material for: Stony coral tissue loss disease indirectly alters reef communities
Source: Sci Adv. 2024 May 3;10(18):eadk6808. doi: 10.1126/sciadv.adk6808 (PMC11068009; doi:10.1126/sciadv.adk6808)
Supplement: Supplementary file 1 — Figs. S1 and S2 Table S1 References [file sciadv.adk6808_sm.pdf]

Science Advances
AAAS

Supplementary Materials for

Stony coral tissue loss disease indirectly alters reef communities

Sara D. Swaminathan *et al.*

Corresponding author: Sara D. Swaminathan, saradeviswaminathan@gmail.com

Sci. Adv. **10**, eadk6808 (2024)
DOI: 10.1126/sciadv.adk6808

The PDF file includes:

Figs. S1 and S2
Table S1
References

Other Supplementary Material for this manuscript includes the following:

Dataset S1

Susceptibility from Brandt (68)	Susceptibility from Estrada-Saldívar et al. (51)	Species name	Grouping category
Resistant	Resistant	*Acropora cervicornis, Acropora palmata, Porites astreoides, Porites divaricata, Porites furcata, Porites porites*	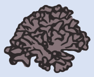 Resistant
Presumed susceptible	Resistant	*Agaricia agaricites, Agaricia fragilis, Agaricia grahamae, Agaricia humilis, Agaricia lamarcki, Agaricia spp., Agaricia undata, Favia fragum, Scolymia cubensis, Scolymia lacera, Scolymia spp., Undaria spp.*	
Resistant	No data	*Oculina diffusa, Oculina spp., Cladocora arbuscula*	
No data	Resistant	*Madracis decactis, Manicina areolata, Branching Porites spp., Siderastrea radians*	
Susceptible	Susceptible	*Colpophyllia natans, Dichocoenia stokesii, Diploria labyrinthiformis, Eusmilia fastigiata, Meandrina meandrites, Meandrina spp., Pseudodiploria strigosa, Pseudodiploria spp.*	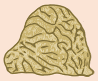 Susceptible
Intermediate	Resistant (n=1)	*Solenastrea bournoni*	
Intermediate	Susceptible	*Montastraea cavernosa, Montastraea spp., Orbicella annularis, Orbicella faveolata, Orbicella franksi, Orbicella spp., Siderastrea siderea, Stephanocoenia intersepta*	
Presumed susceptible	Susceptible	*Agaricia tenuifolia, Helioseris cucullata, Isophyllia rigida, Isophyllia sinuosa, Isophyllia spp., Mycetophyllia aliciae, Mycetophyllia danaana, Mycetophyllia ferox, Mycetophyllia lamarckiana, Mycetophyllia spp.*	
Susceptible	No data	*Dendrogyra cylindrus, Pseudodiploria clivosa*	
Presumed susceptible	No data	*Madracis myriaster, Madracis auretenra, Mussa angulosa*	
No data	No data	Acropora prolifera, Madracis carmabi, Madracis pharensis, Madracis senaria, Madracis formosa, Phyllangia americana, Porites branneri, Porites colonensis, Tubastraea coccinea	Unknown susceptibility
Mixed species	Mixed species	Siderastrea spp., Madracis spp.	

Table S1. SCTLD-susceptibility categories for coral species reported in Brandt (68) in Column 1 and Estrada-Saldívar et al. (51) in Column 2. The “Species name” column lists coral species found in the NCRMP and TCRMP datasets. The “Grouping category” column lists the grouping category used in the present study based on these sources.

Dataset S1. Auxiliary Excel file showing the grouping categories for fish species found in the NCRMP dataset. The datasheet lists the scientific and common names for each species, some additional taxonomic information, and grouping categories based on Strona et al. (40) and FishBase (72). The column “fish category determination” lists the grouping category used in the present study based on these sources.

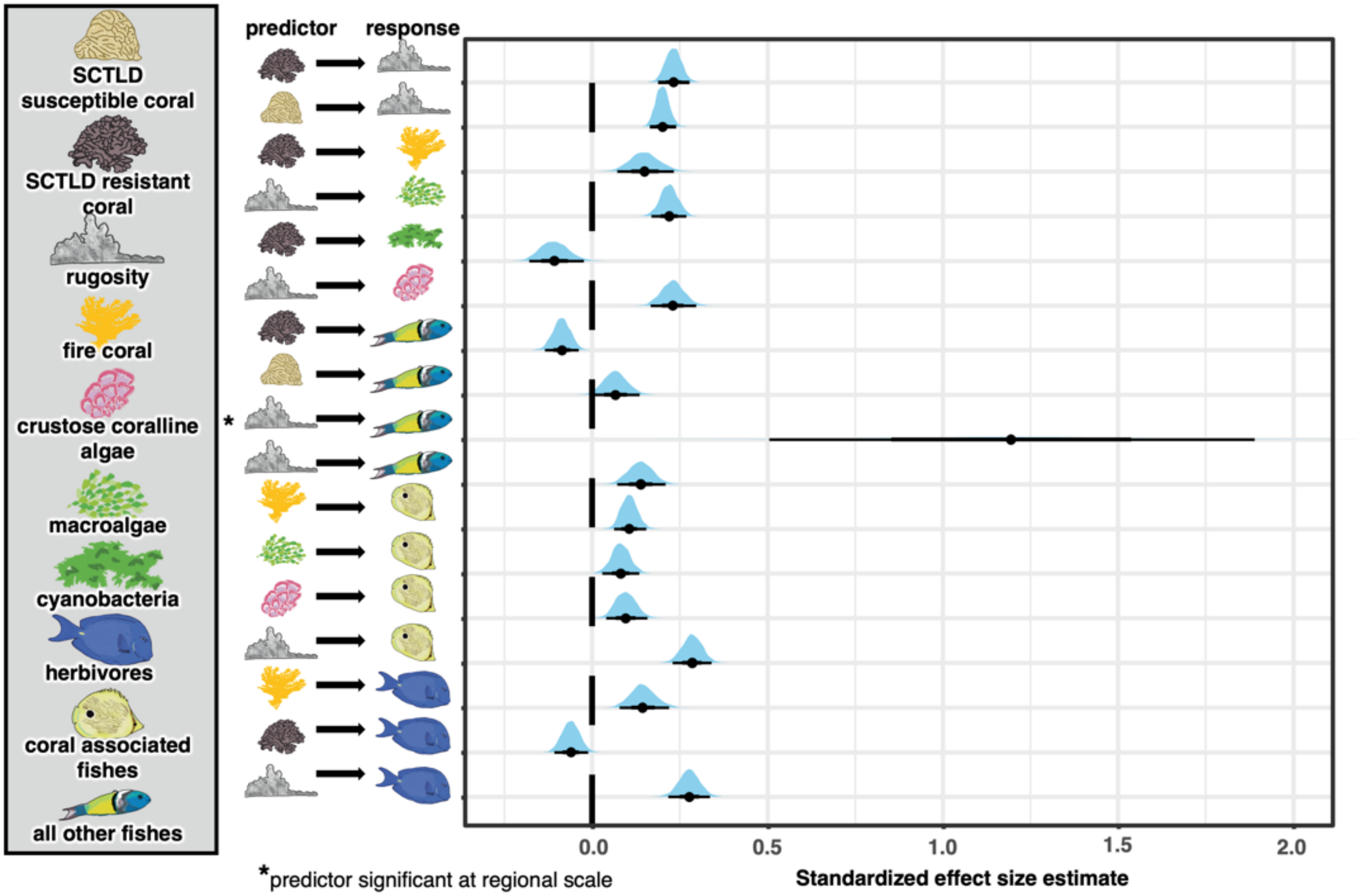

Fig. S1. Coral reef communities consist of numerous relationships between benthic organisms, rugosity, and fish. Results from the Bayesian structural equation model showing posterior distributions of relationships tested in the model where either the site-level group-centered means or subregional means (marked with an asterisk) of the predictors contributed significantly to the response. 95% credible intervals (CIs) are shown by the thin black lines of each stat-eye plot, and 65% CIs are shown by the thicker black lines. The further the CI is from 0, indicated by the dashed black line, the stronger the response to SCTLD. Plots to the right of the dashed line are positive relationships and those to the left show negative relationships.

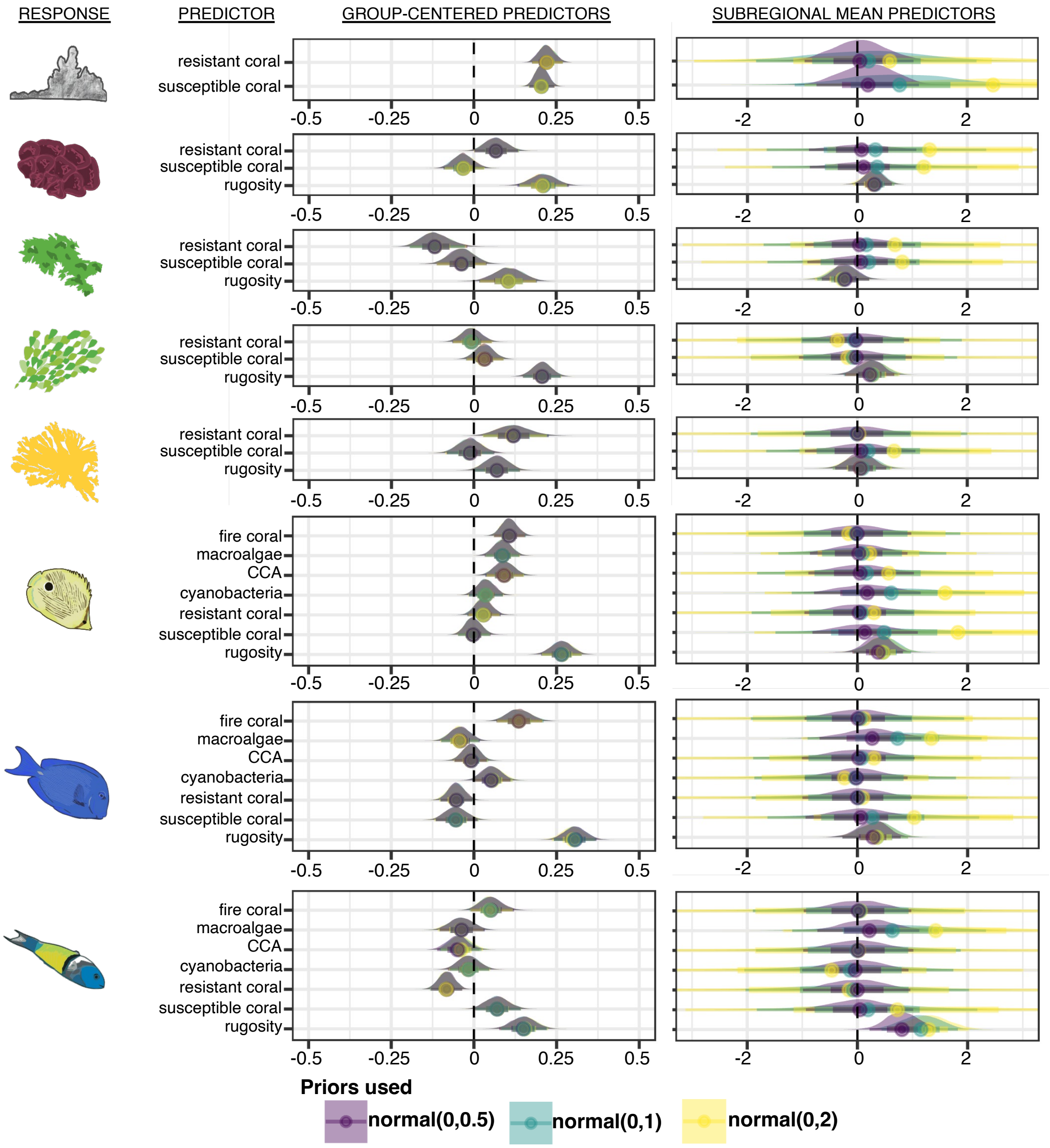

Fig. S2. The priors used for the final SEM were uninformative and did not bias results. Results from a priors sensitivity analysis conducted on the structural equation model (SEM). The icons in the left column represent the response variable (from top-bottom: rugosity, crustose coralline algae, cyanobacteria, macroalgae, fire coral, coral-associated fishes, herbivorous fishes, and all other fishes). The y-axis labels show the predictor variables; with group-centered predictor variables in the left column, and the subregional mean predictor variables in the right column. The stat-eye plots show the posterior distributions of the SEM run separately with three different sets of priors for the slope term: normal(0, 0.5), which are twice as narrow as the original priors, shown in purple; normal(0,1), which are the original priors and those used in the final model, shown in green; and normal(0,2), which are twice as wide as the original priors, shown in yellow. The points at the center of each distribution represent mean effect size estimates.

REFERENCES AND NOTES

1. L. Alvarez-Filip, F. J. González-Barrios, E. Pérez-Cervantes, A. Molina-Hernández, N. Estrada-Saldívar, Stony coral tissue loss disease decimated Caribbean coral populations and reshaped reef functionality. *Commun. Biol.* **5**, 440 (2022).

2. M. E. Brandt, R. S. Ennis, S. S. Meiling, J. Townsend, K. Cobleigh, A. Glahn, J. Quetel, V. Brandtneris, L. M. Henderson, T. B. Smith, The emergence and initial impact of Stony Coral Tissue Loss Disease (SCTLD) in the United States Virgin Islands. *Front. Mar. Sci.* **8**, 715329 (2021).

3. C. J. Walton, N. K. Hayes, D. S. Gilliam, Impacts of a regional, multi-year, multi-species coral disease outbreak in Southeast Florida. *Front. Mar. Sci.* **5**, 323 (2018).

4. W. F. Precht, B. E. Gintert, M. L. Robbart, R. Fura, R. van Woesik, Unprecedented disease-related coral mortality in Southeastern Florida. *Sci. Rep.* **6**, 31374 (2016).

5. J. L. Meyer, J. Castellanos-Gell, G. S. Aeby, C. C. Häse, B. Ushijima, V. J. Paul, Microbial community shifts associated with the ongoing Stony Coral Tissue Loss Disease Outbreak on the Florida Reef Tract. *Front. Microbiol.* **10**, 2244 (2019).

6. C. C. Becker, M. Brandt, C. A. Miller, A. Apprill, Microbial bioindicators of Stony Coral Tissue Loss Disease identified in corals and overlying waters using a rapid field-based sequencing approach. *Environ. Microbiol.* **24**, 1166–1182 (2022).

7. J. Meyer, Development of probiotics and alternative treatments for stony coral tissue loss disease (Florida Department of Environmental Protection, 2021).

8. K. L. Neely, K. A. Macaulay, E. K. Hower, M. A. Dobler, Effectiveness of topical antibiotics in treating corals affected by Stony Coral Tissue Loss Disease. *PeerJ* **8**, e9289 (2020).

9. B. Ushijima, S. P. Gunasekera, J. L. Meyer, J. Tittl, K. A. Pitts, S. Thompson, J. M. Sneed, Y. Ding, M. Chen, L. Jay Houk, G. S. Aeby, C. C. Häse, V. J. Paul, Chemical and genomic characterization of a potential probiotic treatment for stony coral tissue loss disease. *Commun. Biol.* **6**, 248 (2023).

10. K. M. Beavers, E. W. Van Buren, A. M. Rossin, M. A. Emery, A. J. Veglia, C. E. Karrick, N. J. MacKnight, B. A. Dimos, S. S. Meiling, T. B. Smith, A. Apprill, E. M. Muller, D. M. Holstein, A. M. S. Correa, M. E. Brandt, L. D. Mydlarz, Stony coral tissue loss disease induces transcriptional signatures of in situ degradation of dysfunctional Symbiodiniaceae. *Nat. Commun.* **14**, 2915 (2023).

11. T. M. Work, T. M. Weatherby, J. H. Landsberg, Y. Kiryu, S. M. Cook, E. C. Peters, Viral-like particles are associated with endosymbiont pathology in Florida corals affected by Stony Coral Tissue Loss Disease. *Front. Mar. Sci.* **8**, 750658 (2021).

12. K. L. Neely, C. L. Lewis, K. S. Lunz, L. Kabay, Rapid population decline of the pillar coral *Dendrogyra cylindrus* along the Florida Reef Tract. *Front. Mar. Sci.* **8**, 656515 (2021).

13. N. K. Hayes, C. J. Walton, D. S. Gilliam, Tissue loss disease outbreak significantly alters the Southeast Florida stony coral assemblage. *Front. Mar. Sci.* **9**, 975894 (2022).

14. I. Hewson, J. B. Button, B. M. Gudenkauf, B. Miner, A. L. Newton, J. K. Gaydos, J. Wynne, C. L. Groves, G. Hendler, M. Murray, S. Fradkin, M. Breitbart, E. Fahsbender, K. D. Lafferty, A. M. Kilpatrick, C. M. Miner, P. Raimondi, L. Lahner, C. S. Friedman, S. Daniels, M. Haulena, J. Marliave, C. A. Burge, M. E. Eisenlord, C. D. Harvell, Densovirus associated with sea-star wasting disease and mass mortality. *Proc. Natl. Acad. Sci. U.S.A.* **111**, 17278–17283 (2014).

15. C. J. Feehan, R. E. Scheibling, Effects of sea urchin disease on coastal marine ecosystems. *Mar. Biol.* **161**, 1467–1485 (2014).

16. H. A. Lessios, Mass mortality of *Diadema antillarum* in the Caribbean: What have we learned? *Annu. Rev. Ecol. Syst.* **19**, 371–393 (1988).

17. K. D. Lafferty, C. D. Harvell, The role of infectious diseases in marine communities, in *Marine Community Ecology and Conservation* (Sinauer Associates Inc., 2014), pp. 85–108.

18. M. Behrens, K. Lafferty, Effects of marine reserves and urchin disease on southern Californian rocky reef communities. *Mar. Ecol. Prog. Ser.* **279**, 129–139 (2004).

19. M. Reaka-Kudla, The global biodiversity of coral reefs: A comparison with rainforests, in *Biodiversity II: Understanding and Protecting Our Natural Resources* (Joseph Henry/National Academy Press, 1997), pp. 83–108.

20. J. B. C. Jackson, M. X. Kirby, W. H. Berger, K. A. Bjorndal, L. W. Botsford, B. J. Bourque, R. H. Bradbury, R. Cooke, J. Erlandson, J. A. Estes, T. P. Hughes, S. Kidwell, C. B. Lange, H. S. Lenihan, J. M. Pandolfi, C. H. Peterson, R. S. Steneck, M. J. Tegner, R. R. Warner, Historical overfishing and the recent collapse of coastal ecosystems. *Science* **293**, 629–637 (2001).

21. J. B. C. Jackson, Reefs since Columbus. *Coral Reefs* **16**, S23–S32 (1997).

22. W. D. Liddell, S. L. Ohlhorst, Changes in benthic community composition following the mass mortality of *Diadema* at Jamaica. *J. Exp. Mar. Biol. Ecol.* **95**, 271–278 (1986).

23. R. C. Carpenter, Mass mortality of *Diadema antillarum*. *Mar. Biol.* **104**, 67–77 (1990).

24. L. Cheng, K. von Schuckmann, J. P. Abraham, K. E. Trenberth, M. E. Mann, L. Zanna, M. H. England, J. D. Zika, J. T. Fasullo, Y. Yu, Y. Pan, J. Zhu, E. R. Newsom, B. Bronselaer, X. Lin, Past and future ocean warming. *Nat. Rev. Earth Environ.* **3**, 776–794 (2022).

25. A. C. Baker, P. W. Glynn, B. Riegl, Climate change and coral reef bleaching: An ecological assessment of long-term impacts, recovery trends and future outlook. *Estuar. Coast. Shelf Sci.* **80**, 435–471 (2008).

26. V. Schoepf, A. G. Grottoli, S. J. Levas, M. D. Aschaffenburg, J. H. Baumann, Y. Matsui, M. E. Warner, Annual coral bleaching and the long-term recovery capacity of coral. *Proc. Biol. Sci.* **282**, 20151887 (2015).

27. R. B. Aronson, W. F. Precht, White-band disease and the changing face of Caribbean coral reefs. *Hydrobiologia* **460**, 25–38 (2001).

28. K. G. Kuta, L. L. Richardson, Abundance and distribution of black band disease on coral reefs in the northern Florida Keys. *Coral Reefs* **15**, 219–223 (1996).

29. A. W. Bruckner, R. J. Bruckner, Consequences of yellow band disease (YBD) on *Montastraea annularis* (species complex) populations on remote reefs off Mona Island, Puerto Rico. *Dis. Aquat. Organ.* **69**, 67–73 (2006).

30. K. D. Lafferty, J. W. Porter, S. E. Ford, Are diseases increasing in the ocean? *Annu. Rev. Ecol. Evol. Syst.* **35**, 31–54 (2004).

31. K. E. Kovalenko, S. M. Thomaz, D. M. Warfe, Habitat complexity: Approaches and future directions. *Hydrobiologia* **685**, 1–17 (2012).

32. D. J. Coker, S. K. Wilson, M. S. Pratchett, Importance of live coral habitat for reef fishes. *Rev. Fish Biol. Fish.* **24**, 89–126 (2014).

33. E. S. Darling, N. A. J. Graham, F. A. Januchowski-Hartley, K. L. Nash, M. S. Pratchett, S. K. Wilson, Relationships between structural complexity, coral traits, and reef fish assemblages. *Coral Reefs* **36**, 561–575 (2017).

34. A. Rogers, J. L. Blanchard, S. P. Newman, C. S. Dryden, P. J. Mumby, High refuge availability on coral reefs increases the vulnerability of reef-associated predators to overexploitation. *Ecology* **99**, 450–463 (2018).

35. A. R. Harborne, A. Rogers, Y.-M. Bozec, P. J. Mumby, Multiple stressors and the functioning of coral reefs. *Ann. Rev. Mar. Sci.* **9**, 445–468 (2017).

36. M. A. Hixon, J. P. Beets, Predation, prey refuges, and the structure of coral-reef fish assemblages. *Ecol. Monogr.* **63**, 77–101 (1993).

37. S. J. Brandl, M. J. Emslie, D. M. Ceccarelli, Z. T. Richards, Habitat degradation increases functional originality in highly diverse coral reef fish assemblages. *Ecosphere* **7**, e01557 (2016).

38. D. A. Feary, G. R. Almany, M. I. McCormick, G. P. Jones, Habitat choice, recruitment and the response of coral reef fishes to coral degradation. *Oecologia* **153**, 727–737 (2007).

39. G. R. Russ, J. R. Rizzari, R. A. Abesamis, A. C. Alcala, Coral cover a stronger driver of reef fish trophic biomass than fishing. *Ecol. Appl.* **31**, e02224 (2021).

40. G. Strona, K. D. Lafferty, S. Fattorini, P. S. A. Beck, F. Guilhaumon, R. Arrigoni, S. Montano, D. Seveso, P. Galli, S. Planes, V. Parravicini, Global tropical reef fish richness could decline by around half if corals are lost. *Proc. Biol. Sci.* **288**, 20210274 (2021).

41. M. L. Reaka-Kudla, J. S. Feingold, W. Glynn, Experimental studies of rapid bioerosion of coral reefs in the Galápagos Islands. *Coral Reefs* **15**, 101–107 (1996).

42. B. Gratwicke, M. R. Speight, The relationship between fish species richness, abundance and habitat complexity in a range of shallow tropical marine habitats. *J. Fish Biol.* **66**, 650–667 (2005).

43. B. Gratwicke, M. Speight, Effects of habitat complexity on Caribbean marine fish assemblages. *Mar. Ecol. Prog. Ser.* **292**, 301–310 (2005).

44. A. R. Harborne, P. J. Mumby, R. Ferrari, The effectiveness of different meso-scale rugosity metrics for predicting intra-habitat variation in coral-reef fish assemblages. *Environ. Biol. Fishes* **94**, 431–442 (2012).

45. C. I. Elliff, I. R. Silva, Coral reefs as the first line of defense: Shoreline protection in face of climate change. *Mar. Environ. Res.* **127**, 148–154 (2017).

46. M. Spalding, L. Burke, S. A. Wood, J. Ashpole, J. Hutchison, P. zu Ermgassen, Mapping the global value and distribution of coral reef tourism. *Mar. Policy* **82**, 104–113 (2017).

47. H. Cesar, L. Burke, L. Pet-Soede, The economics of worldwide coral reef degradation (International Coral Reef Action Network, 2003).

48. R. S. Ennis, S. L. Heidmann, L. M. Henderson, M. Warham, T. B. Smith, The United States Virgin Islands Territorial Coral Reef Monitoring Program (2021); https://sites.google.com/site/usvitcrmp/tcrmp-reports?authuser=0.

49. NOAA National Centers for Environmental Information, *National Coral Reef Monitoring Program: Benthic, Coral Demography, and Reef Fish Visual Census data* (2021).

50. AGRRA, Map of coral cover of susceptible coral species to SCTLD (2023); www.agrra.org.

51. N. Estrada-Saldívar, B. A. Quiroga-García, E. Pérez-Cervantes, O. O. Rivera-Garibay, L. Alvarez-Filip, Effects of the Stony Coral Tissue Loss Disease outbreak on coral communities and the benthic composition of Cozumel reefs. *Front. Mar. Sci.* **8**, 632777 (2021).

52. S. S. Meiling, E. M. Muller, D. Lasseigne, A. Rossin, A. J. Veglia, N. MacKnight, B. Dimos, N. Huntley, A. M. S. Correa, T. B. Smith, D. M. Holstein, L. D. Mydlarz, A. Apprill, M. E. Brandt, Variable species responses to experimental stony coral tissue loss disease (SCTLD) exposure. *Front. Mar. Sci.* **8**, 670829 (2021).

53. J. M. Sneed, K. H. Sharp, K. B. Ritchie, V. J. Paul, The chemical cue tetrabromopyrrole from a biofilm bacterium induces settlement of multiple Caribbean corals. *Proc. Biol. Sci.* **281**, 20133086 (2014).

54. M. J. A. Vermeij, M. L. Dailer, C. M. Smith, Crustose coralline algae can suppress macroalgal growth and recruitment on Hawaiian coral reefs. *Mar. Ecol. Prog. Ser.* **422**, 1–7 (2011).

55. T. P. Hughes, Catastrophes, phase shifts, and large-scale degradation of a Caribbean coral reef. *Science* **265**, 1547–1551 (1994).

56. J. L. Meyer, S. P. Gunasekera, R. M. Scott, V. J. Paul, M. Teplitski, Microbiome shifts and the inhibition of quorum sensing by Black Band Disease cyanobacteria. *ISME J.* **10**, 1204–1216 (2016).

57. P. Copper, Ancient reef ecosystem expansion and collapse. *Coral Reefs* **13**, 3–11 (1994).

58. N. A. J. Graham, K. L. Nash, The importance of structural complexity in coral reef ecosystems. *Coral Reefs* **32**, 315–326 (2013).

59. I. C. S. Leal, M. E. de Araújo, S. R. da Cunha, P. H. C. Pereira, The influence of fire-coral colony size and agonistic behaviour of territorial damselfish on associated coral reef fish communities. *Mar. Environ. Res.* **108**, 45–54 (2015).

60. G. Roff, J. Zhao, P. J. Mumby, Decadal-scale rates of reef erosion following El Niño-related mass coral mortality. *Glob. Chang. Biol.* **21**, 4415–4424 (2015).

61. J. Morais, R. Morais, S. B. Tebbett, D. R. Bellwood, On the fate of dead coral colonies. *Funct. Ecol.* **36**, 3148–3160 (2022).

62. A. Molina-Hernández, F. Medellín-Maldonado, I. D. Lange, C. T. Perry, L. Álvarez-Filip, Coral reef erosion: In situ measurement on different dead coral substrates on a Caribbean reef. *Limnol. Oceanogr.* **67**, 2734–2749 (2022).

63. Y.-M. Bozec, L. Alvarez-Filip, P. J. Mumby, The dynamics of architectural complexity on coral reefs under climate change. *Glob. Chang. Biol.* **21**, 223–235 (2015).

64. A. C. Siqueira, P. Muruga, D. R. Bellwood, On the evolution of fish–coral interactions. *Ecol. Lett.* **26**, 1348–1358 (2023).

65. K. R. Noonan, M. J. Childress, Association of butterflyfishes and stony coral tissue loss disease in the Florida Keys. *Coral Reefs* **39**, 1581–1590 (2020).

66. R Core Team, *R: A Language and Environment for Statistical Computing, version 4.2.2* (R Foundation for Statistical Computing, 2022); www.R-project.org/.

67. R. S. Ennis, S. L. Heidmann, L. M. Henderson, M. Warham, T. B. Smith, The United States Virgin Islands Territorial Coral Reef Monitoring Program (2021); https://sites.google.com/site/usvitcrmp/tcrmp-reports?authuser=0.

68. M. E. Brandt, Stony Coral Tissue Loss Disease (SCTLD) case definition (Florida Department of Environmental Protection, 2018); https://floridadep.gov/rcp/coral/documents/stony-coral-tissue-loss-disease-sctld-case-definition.

69. R. McElreath, *Statistical Rethinking: A Bayesian Course with Examples in R and Stan* (CRC Press/Taylor & Francis Group, 2016), Chapman & Hall/CRC Texts in Statistical Science Series.

70. NOAA National Centers for Environmental Information, *Coral Reef Conservation Program Documentation for NOAA's Coral Reef Conservation Program (CRCP) National Coral Reef Monitoring Program (NCRMP) data archived at NCEI*. Dataset, NCEI Accession 0157633 (2016); www.ncei.noaa.gov/archive/accession/0157633.

71. Y. Mundlak, On the pooling of time series and cross section data. *Econometrica* **46**, 69–85 (1978).

72. C. Boettiger, D. T. Lang, P. C. Wainwright, rfishbase: Exploring, manipulating and visualizing FishBase data from R. *J. Fish Biol.* **81**, 2030–2039 (2012).

73. A. J. Cole, M. S. Pratchett, G. P. Jones, Diversity and functional importance of coral-feeding fishes on tropical coral reefs. *Fish Fish.* **9**, 286–307 (2008).

74. K. L. Nash, N. A. J. Graham, S. Jennings, S. K. Wilson, D. R. Bellwood, Herbivore cross-scale redundancy supports response diversity and promotes coral reef resilience. *J. Appl. Ecol.* **53**, 646–655 (2016).

75. N. A. J. Graham, P. Chabanet, R. D. Evans, S. Jennings, Y. Letourneur, M. Aaron MacNeil, T. R. McClanahan, M. C. Öhman, N. V. C. Polunin, S. K. Wilson, Extinction vulnerability of coral reef fishes. *Ecol. Lett.* **14**, 341–348 (2011).

76. K. Thulasiraman, M. N. S. Swamy, 5.7 acyclic directed graphs, in *Graphs: Theory and Algorithms*, 118-119 (Wiley, 1992).

77. C. E. L. Ferreira, J. E. A. Goncalves, R. Coutinho, Community structure of fishes and habitat complexity on a tropical rocky shore. *Environ. Biol. Fishes* **61**, 353–369 (2001).

78. A. M. Canterle, L. T. Nunes, L. Fontoura, H. A. Maia, S. R. Floeter, Reef microhabitats mediate fish feeding intensity and agonistic interactions at Príncipe Island Biosphere Reserve, Tropical Eastern Atlantic. *Mar. Ecol.* **41**, e12609 (2020).

79. K. M. Chong-Seng, T. D. Mannering, M. S. Pratchett, D. R. Bellwood, N. A. J. Graham, The influence of coral reef benthic condition on associated fish assemblages. *PLOS ONE* **7**, e42167 (2012).

80. National Centers for Coastal Ocean Science (NCCOS), NOAA National Centers for Environmental Information, *National Coral Reef Monitoring Program: Assessment of Coral Reef Benthic Communities in the U.S. Virgin Islands* (2016); https://doi.org/10.7289/V5WW7FQK.

81. National Centers for Coastal Ocean Science (NCCOS), Southeast Fisheries Science Center (SEFSC), NOAA National Centers for Environmental Information, *National Coral Reef Monitoring Program: Assessment of coral reef benthic communities in Puerto Rico* (2018); https://doi.org/10.7289/V5PG1Q23.

82. NOAA Southeast Fisheries Science Center, NOAA National Centers for Coastal Ocean Science, NOAA National Centers for Environmental Information, *National Coral Reef Monitoring Program: Assessment of Coral Reef Benthic Communities in the Florida Reef Tract* (2018); https://doi.org/10.7289/v5xw4h4z.

83. National Centers for Coastal Ocean Science (NCCOS), NOAA National Centers for Environmental Information, *National Coral Reef Monitoring Program: Assessment of Coral Reef Fish Communities in the U.S. Virgin Islands* (2016); https://doi.org/10.7289/V5F769MM.

84. National Centers for Coastal Ocean Science (NCCOS), Southeast Fisheries Science Center (SEFSC), NOAA National Centers for Environmental Information, *National Coral Reef Monitoring Program: Assessment of Coral Reef Fish Communities in Puerto Rico* (2018); https://doi.org/10.7289/V5T72FRZ.

85. NOAA Southeast Fisheries Science Center, NOAA National Centers for Coastal Ocean Science, NOAA National Centers for Environmental Information, *National Coral Reef Monitoring Program: Assessment of Coral Reef Fish Communities in the Florida Reef Tract* (2018); https://doi.org/10.7289/v52n50ks.

86. WoRMS Editorial Board (2024). World Register of Marine Species, https://www.marinespecies.org at VLIZ [accessed 23 March 2024] doi:10.14284/170.
